# Supplementary material for: Short-Term Exposure to Fine Particulate Matter and Hospitalizations for Acute Lower Respiratory Infection in Korean Children: A Time-Series Study in Seven Metropolitan Cities
Source: Int J Environ Res Public Health. 2020 Dec 28;18(1):144. doi: 10.3390/ijerph18010144 (PMC7795137; doi:10.3390/ijerph18010144)
Supplement: Supplementary file 1 [file ijerph-18-00144-s001.pdf]

*Supplementary Materials*

# Short-term exposure to fine particulate matter and hospitalizations for acute lower respiratory infection in Korean Children: a time-series study in 7 metropolitan cities.

**Supplementary explanations**

**Table 1.** Daily $PM_{2.5}$ estimated using CMAQ.

Daily $PM_{2.5}$ concentration was estimated with meteorology inputs simulated from a Weather Research and Forecasting (WRF, version 3.3.1) model and anthropogenic emissions processed through the Sparse Matrix Operator Kernel Emission (SMOKE, version 3.1) [1-3]. For the early stages of weather conditions, National Center for Environmental Prediction/Final Analysis (NCEP-FNL), a reanalysis data from the U.S. Oceanic and Atmospheric Administration (NOAA), was used. The Model of Emissions of Gases and Aerosols from Nature (MEGAN, version 2.04) was used to estimate emissions of biogenic volatile organic compounds. To simulate the secondary aerosol formation and growth, and gas-phase chemical reactions, the 5th generation CMAQ Aerosol Module (AERO5) and the statewide air pollution research center version 99 (SAPRC99) were used for the aerosol and chemical mechanisms, respectively [1-3]. For air quality simulation, two model domains were used for further analysis, with horizontal grid resolutions of 27 km (covering Northeast Asia) to include regional transports of $PM_{2.5}$ and its precursors, and 9 km (South Korea) to prepare the nationwide $PM_{2.5}$ at a finer grid resolution. Having the air quality simulation platform ready, we first simulated the hourly concentration of $PM_{2.5}$ at each grid and resampled the values to the Earth level based on the GIS shape-file of Korea [4]. Our exposure data are data of spatial interpolation results based on observational data. The observational data are monitored data provided by the Ministry of Environment. We considered factors that affect atmospheric concentrations such as emissions, weather condition, and topography for space interpolation. But, there are some missing points in the spatial and temporal aspects of the observational data. In particular, South Korea has many coastal and islands because it is a peninsula surrounded by the sea. In coastal or islands, there is no data to interpolate. Instead, we used the simulation results. We've interpolated the space by the hour. Domestic emission data used Clean Air Policy Support System (CAPSS) 2010 and overseas emission data used MICS-Asia 2010 (Model Inter-Comparison Study for Asia). CAPSS is a list of annual national emissions provided by the National Institute of Environmental Research. In conclusion, we used the exposure data fused with the WRF and CMAQ simulation based on the observational data.

**Equations described in the manuscript**

The apparent temperature simple calculation equation is as follows [5, 6].

Equation (1)

Apparent temperature (°C) = −2.653 + (0.994 × Temperature (°C)) + (0.0153 × Dew point temperature $(°C)^2$)   (1)

## Supplementary tables

**Table S1.** The association between single lag $PM_{2.5}$ concentrations and ALRI hospital admissions among children the seven major cities in Korea, 2018-2016.

| | **Cities** | | | | | | | | | | | | | | | |
|---|---|---|---|---|---|---|---|---|---|---|---|---|---|---|---|---|
| | Total | | Seoul | | Busan | | Daegu | | Incheon | | Gwangju | | Daejeon | | Ulsan | |
| Sing lag | Percentage change | 95% CI | Percentage change | 95% CI | Percentage change | 95% CI | Percentage change | 95% CI | Percentage change | 95% CI | Percentage change | 95% CI | Percentage change | 95% CI | Percentage change | 95% CI |
| 0 | **0.58** | **(0.41, 0.76)** | **0.58** | (0.25, 0.92) | **0.77** | (0.41, 1.12) | **0.56** | (0.05, 1.08) | 0.53 | (-0.05, 1.11) | 0.40 | (-0.06, 0.86) | 0.54 | (-0.19, 1.28) | 0.46 | (-0.21, 1.13) |
| 1 | **0.41** | **(0.19, 0.63)** | **0.81** | (0.47, 1.15) | **0.48** | (0.13, 0.84) | 0.17 | (-0.35, 0.69) | 0.33 | (-0.24, 0.91) | **0.46** | (0.01, 0.91) | 0.03 | (-0.70, 0.77) | -0.14 | (-0.82, 0.54) |
| 2 | **0.57** | **(0.40, 0.74)** | **0.69** | (0.36, 1.03) | **0.64** | (0.29, 0.98) | **0.82** | (0.32, 1.32) | **0.59** | (0.03, 1.16) | 0.20 | (-0.24, 0.65) | 0.08 | (-0.64, 0.81) | 0.58 | (-0.08, 1.23) |
| 3 | **0.42** | **(0.11, 0.73)** | **0.52** | (0.20, 0.84) | -0.07 | (-0.42, 0.27) | **0.61** | (0.12, 1.11) | 0.03 | (-0.52, 0.58) | 0.08 | (-0.36, 0.52) | **1.04** | (0.35, 1.74) | **1.12** | (0.47, 1.77) |
| 4 | **0.46** | **(0.13, 0.79)** | **0.36** | (0.04, 0.69) | 0.15 | (-0.19, 0.50) | **0.55** | (0.05, 1.04) | 0.08 | (-0.47, 0.64) | 0.01 | (-0.43, 0.46) | **1.23** | (0.53, 1.93) | **1.28** | (0.64, 1.93) |
| 5 | 0.23 | (-0.04, 0.49) | **0.39** | (0.07, 0.72) | -0.06 | (-0.41, 0.29) | 0.38 | (-0.12, 0.88) | -0.25 | (-0.81, 0.30) | -0.10 | (-0.55, 0.34) | **0.98** | (0.27, 1.69) | 0.60 | (-0.05, 1.25) |
| 6 | 0.16 | (-0.04, 0.36) | 0.18 | (-0.15, 0.50) | 0.03 | (-0.32, 0.38) | 0.35 | (-0.14, 0.85) | 0.13 | (-0.42, 0.68) | -0.16 | (-0.60, 0.29) | **0.87** | (0.18, 1.57) | 0.12 | (-0.54, 0.77) |
| 7 | -0.01 | (-0.18, 0.16) | 0.13 | (-0.19, 0.45) | -0.18 | (-0.52, 0.16) | -0.12 | (-0.61, 0.37) | -0.16 | (-0.70, 0.39) | -0.05 | (-0.49, 0.40) | 0.43 | (-0.27, 1.13) | 0.17 | (-0.46, 0.81) |

**Scheme 0.** days before. Time-series model adjusted for same covariates (time, AT, day of weeks). The percentage change is calculated as the change per $PM_{2.5}$ 10 μg/m$^3$ increase.* The bold text means statistically significant.

**Table S2.** The association between moving average $PM_{2.5}$ concentrations and ALRI hospital admissions among children the seven major cities in Korea, 2018-2016. The moving average is the average from the current day to the previous day. Time-series model adjusted for same covariates (time, AT, day of weeks). The percentage change is calculated as the change per $PM_{2.5}$ 10 $\mu g/m^3$ increase.

| | Cities | | | | | | | | | | | | | | | |
|---|---|---|---|---|---|---|---|---|---|---|---|---|---|---|---|---|
| | Total | | Seoul | | Busan | | Daegu | | Incheon | | Gwangju | | Daejeon | | Ulsan | |
| Moving average | Percentage change | 95% CI | Percentage change | 95% CI | Percentage change | 95% CI | Percentage change | 95% CI | Percentage change | 95% CI | Percentage change | 95% CI | Percentage change | 95% CI | Percentage change | 95% CI |
| 01 | **0.68** | **(0.47, 0.88)** | **0.91** | (0.52, 1.30) | **0.83** | (0.42, 1.25) | 0.49 | (-0.11, 1.09) | 0.56 | (-0.12, 1.23) | **0.56** | (0.03, 1.09) | 0.36 | (-0.47, 1.20) | 0.22 | (-0.57, 1.01) |
| 02 | **0.90** | **(0.66, 1.12)** | **1.15** | (0.72, 1.59) | **1.07** | (0.61, 1.54) | **0.90** | (0.23, 1.58) | **0.80** | (0.04, 1.56) | 0.56 | (-0.03, 1.15) | 0.33 | (-0.60, 1.26) | 0.53 | (-0.35, 1.43) |
| 03 | **0.98** | **(0.72, 1.23)** | **1.31** | (0.83, 1.79) | **0.90** | (0.39, 1.41) | **1.15** | (0.40, 1.89) | 0.71 | (-0.12, 1.54) | 0.52 | (-0.13, 1.17) | 0.83 | (-0.19, 1.85) | **1.10** | (0.12, 2.08) |
| 04 | **1.09** | **(0.79, 1.38)** | **1.40** | (0.88, 1.93) | **0.90** | (0.35, 1.46) | **1.36** | (0.55, 2.17) | 0.69 | (-0.20, 1.59) | 0.46 | (-0.23, 1.16) | **1.35** | (0.25, 2.46) | **1.70** | (0.64, 2.77) |
| 05 | **1.14** | **(0.74, 1.54)** | **1.54** | (0.98, 2.10) | **0.83** | (0.24, 1.43) | **1.50** | (0.63, 2.38) | 0.53 | (-0.43, 1.49) | 0.38 | (-0.37, 1.13) | **1.74** | (0.56, 2.93) | **1.92** | (0.78, 3.07) |
| 06 | **1.20** | **(0.71, 1.71)** | **1.60** | (1.00, 2.20) | **0.81** | (0.17, 1.45) | **1.64** | (0.70, 2.58) | 0.57 | (-0.45, 1.61) | 0.28 | (-0.51, 1.08) | **2.09** | (0.83, 3.37) | **1.92** | (0.71, 3.15) |
| 07 | **1.17** | **(0.67, 1.68)** | **1.65** | (1.01, 2.29) | **0.69** | (0.02, 1.37) | **1.54** | (0.55, 2.54) | 0.48 | (-0.61, 1.59) | 0.23 | (-0.61, 1.08) | **2.24** | (0.90, 3.60) | **1.98** | (0.69, 3.29) |

* The bold text means statistically significant.

## Supplementary figures

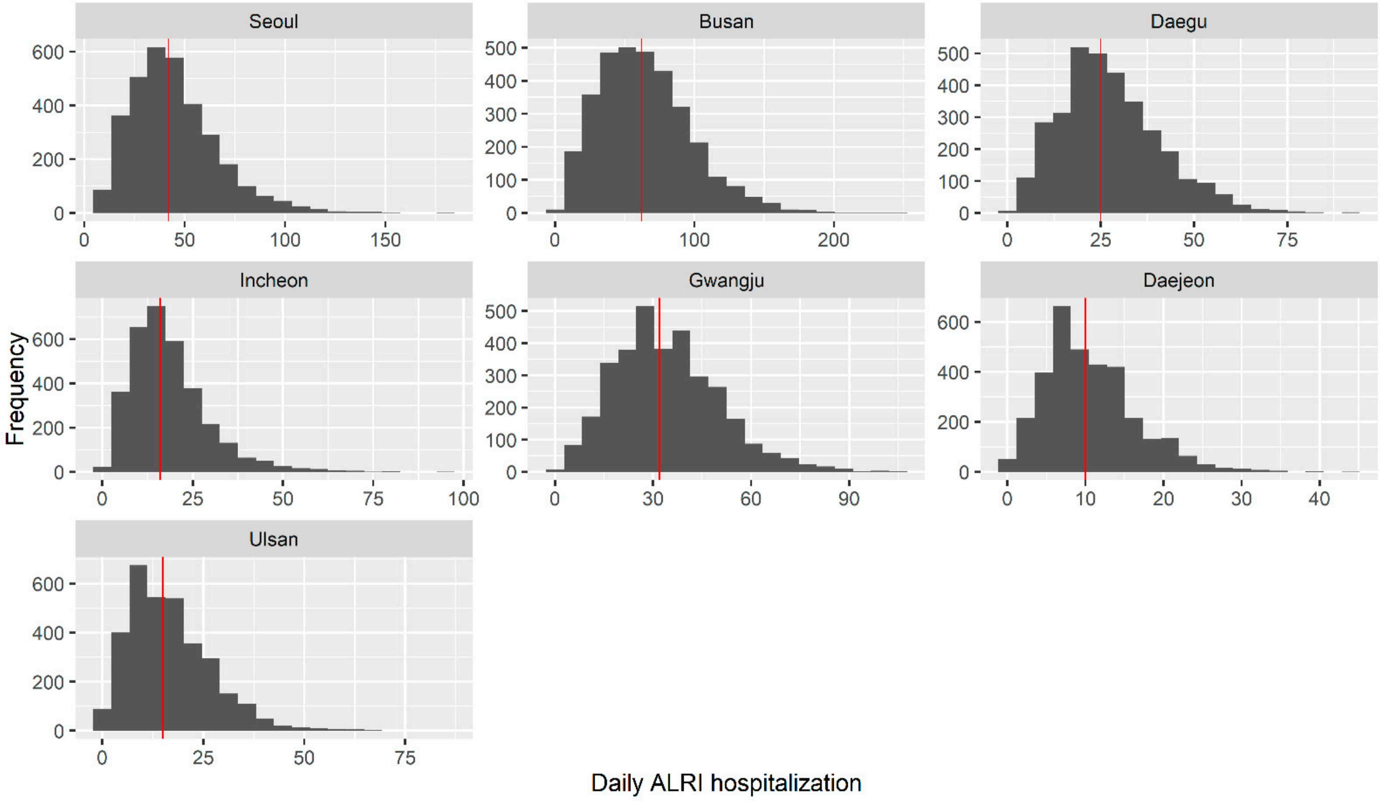


**Figure S1.** The histogram of daily ALRI hospitalization in seven cities, in Korea.

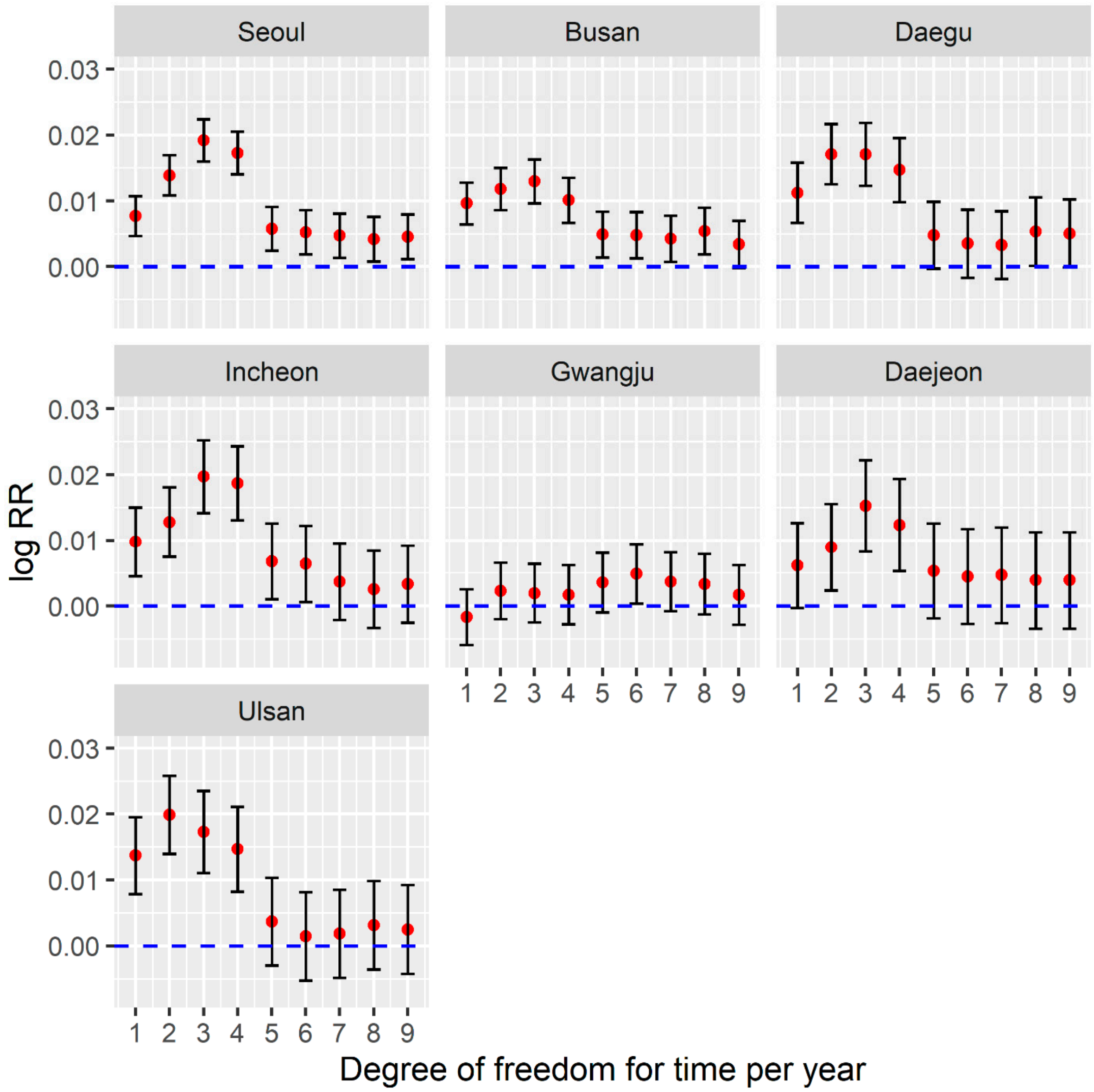


**Figure S2.** Estimation of log relative risk according to the degrees of freedom for time per year. The model was adjusted for apparent temperature, day of weeks and time trends.

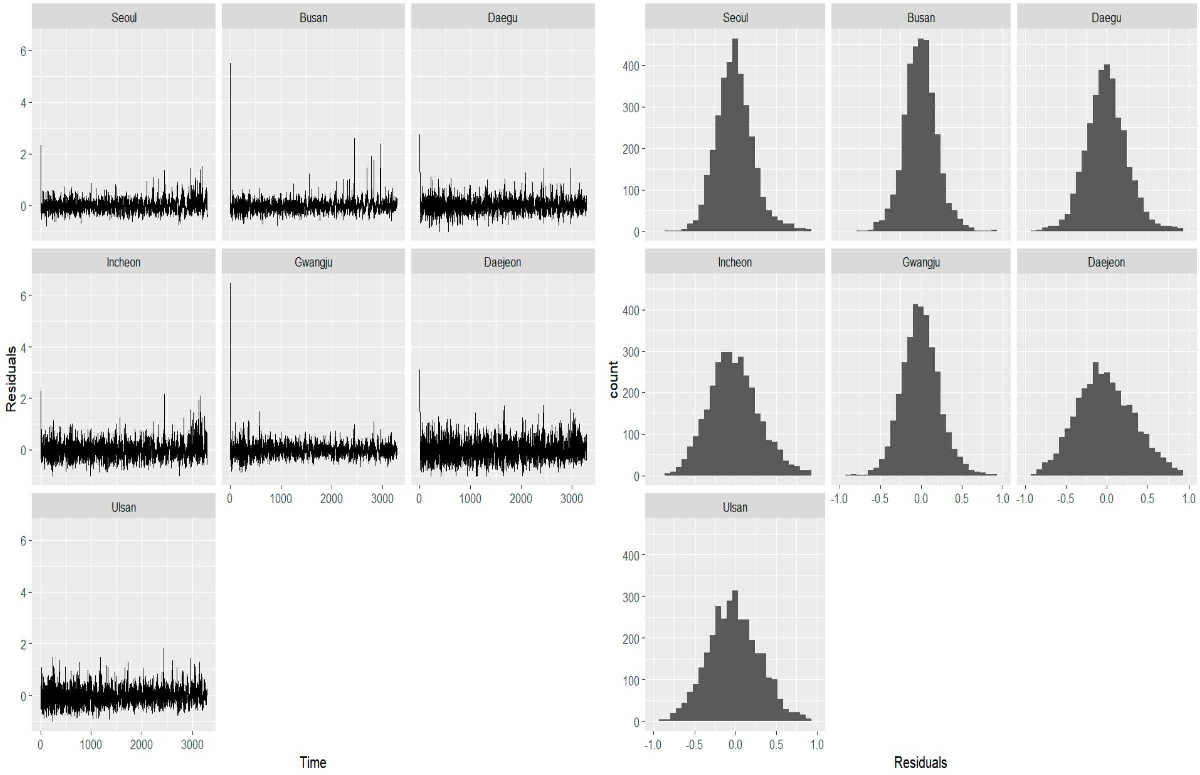


**Figure S3.** Residuals plots for fitted models for each region (left), and residual histograms for fitted models for each region (right).

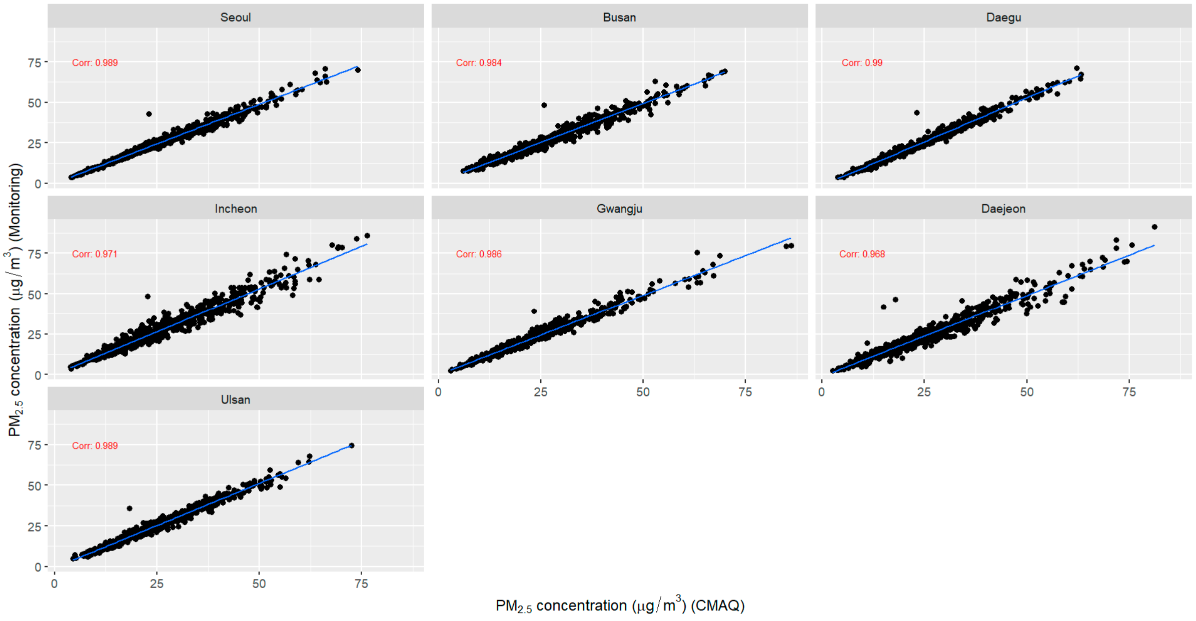


**Figure S4.** Correlation between daily CMAQ $PM_{2.5}$ concentration and Monitoring $PM_{2.5}$ concentration by region (2015-2016).

## References

1. Han, C.; Kim, S.; Lim, Y. H.; Bae, H. J.; Hong, Y. C., Spatial and Temporal Trends of Number of Deaths Attributable to Ambient PM2.5 in the Korea. *J Korean Med Sci* **2018,** 33, (30).
2. Kim, B.-U.; Bae, C.; Kim, H. C.; Kim, E.; Kim, S., Spatially and chemically resolved source apportionment analysis: Case study of high particulate matter event. *Atmos Environ* **2017,** 162, 55-70.
3. Kim, H. C.; Kim, E.; Bae, C.; Cho, J. H.; Kim, B.-U.; Kim, S., Regional contributions to particulate matter concentration in the Seoul metropolitan area, South Korea: seasonal variation and sensitivity to meteorology and emissions inventory. *Atmos Chem Phys* **2017,** 17, (17), 10315-10332.
4. Han, C.; Oh, J.; Lim, Y. H.; Kim, S.; Hong, Y. C., Long-term exposure to fine particulate matter and development of chronic obstructive pulmonary disease in the elderly. *Environ Int* **2020,** 143, 105895.
5. Ng, C. F.; Ueda, K.; Ono, M.; Nitta, H.; Takami, A., Characterizing the effect of summer temperature on heatstroke-related emergency ambulance dispatches in the Kanto area of Japan. *Int J Biometeorol* **2014,** 58, (5), 941-8.
6. Nguyen, J. L.; Schwartz, J.; Dockery, D. W., The relationship between indoor and outdoor temperature, apparent temperature, relative humidity, and absolute humidity. *Indoor Air* **2014,** 24, (1), 103-12.
